# Knowledge, Attitude, and Prevention Practices towards Common Zoonotic Diseases in and around Bahir Dar City, North Western Ethiopia

**DOI:** 10.1155/2024/6642766

**Published:** 2024-03-20

**Authors:** Kassa Demelash Alemayehu, Birhan Agmas Mitiku, Yeshwas Ferede Alemu, Tsegaye Birtualem Nibret

**Affiliations:** ^1^Bahir Dar Poly Technique College, Bahir Dar, Ethiopia; ^2^Department of Veterinary Science, College of Agriculture and Environmental Sciences, Bahir Dar University, Bahir Dar, Ethiopia; ^3^Ethiopian Agriculture Authority, Addis Ababa, Ethiopia

## Abstract

The problem of zoonoses diseases is a global public and veterinary health concern. Globally coordinated and well-established research efforts are essential to successfully fighting and reducing the health burden of zoonoses. In our study area, the interplay of intense livestock animals, agricultural activities, and poor health services characterized the high risks of zoonotic diseases. Thus, people suffer from easily preventable diseases with hygiene and good-quality food. The main objectives of this study were to: (i) evaluate the knowledge, attitude, and prevention practices of people handling farm animals and their products toward common zoonotic diseases; and (ii) estimate the associated risk factors influencing their knowledge, attitude, and prevention practices. A questionnaire-based cross-sectional study was conducted from December 2021 to August 2022. A simple random sampling technique was followed to select respondents. Multivariable logistic regression model analysis was conducted using STATA version 14. The overall level of knowledge, attitude, and prevention practice for the major zoonotic disease was 52.5%, 68.6%, and 39.4%, respectively. Rabies, tuberculosis, taeniasis, anthrax, and brucellosis were the major zoonotic diseases selected by respondents in the study areas. The age of respondents, training status, educational status, and farm location were significantly (*P*  < 0.05) associated with the level of knowledge and prevention practice against zoonotic diseases. This study revealed that the level of knowledge, attitude, and prevention practices for major zoonotic diseases in Bahir Dar City were relatively poor. Therefore, a concerted effort among various government and nongovernment stakeholders, including veterinarians, public health officials, and environmental experts, is needed to create and raise awareness among livestock producers about the transmission and control methods and the economic and public health importance of zoonotic diseases.

## 1. Background and Justification

Livestock performs multiple functions, such as being used as food, input for crop production and soil fertility management, raw material for industry, and cash income, as well as promoting saving, fuel, social functions, and employment [1, 2]. However, when a person works in livestock production, hazards that cause health problems in the working condition may cause sickness, impaired health, and well-being [3].

According to Taylor et al. [4], a comprehensive literature review globally identified that 868 of 1,415 (61%) known human pathogens and 132 of 175 (75%) emerging diseases that affect humans are zoonotic. Across the globe, zoonoses are most impactful on poor livestock workers and have caused an estimated 2.4 billion cases of illness and 2.7 million deaths in humans per year [5]. Today, in both developing and developed countries, a number of new zoonoses have emerged [6].

In Ethiopia, a large proportion of the population is exposed to zoonotic infection because 80% of people depend on agriculture and all the agricultural activities done by livestock or in regular contact with domestic animals, creating an opportunity for infection and the spread of disease [7, 8]. In addition, there has been a growing demand for animal products in many urban and peri-urban communities in low- and middle-income countries (LMIC) that are exposed to food-borne zoonosis. Moreover; animal production in densely populated areas has intensified, which has raised the danger of zoonotic disease infections in humans [9].

In developing countries there is a higher risk of zoonotic disease outbreaks causing high morbidity and mortality, because of several factors. Poor, unsanitary living conditions in close contact with animals, combined with limited understanding of the role of domestic animals and their byproducts in the transmission of zoonotic diseases [10] are major drivers. In addition, a weak collaboration between medical and veterinary professionals, an absence of health education programs, inadequate health service coverage, and inadequate health policies underdiagnosed, underreported, and complex diagnostic tests to confirm their presence are other challenges in developing countries [11, 12].

The awareness of the occupational community towards zoonotic diseases plays an important role in the life cycle and routes of transmission of these diseases to the different arrays of their hosts; the risk factors, prevention, and control of zoonotic diseases are crucial steps toward the development and implementation of appropriate disease prevention and control strategies [13, 14].

In Ethiopia, regarding knowledge, attitude, and prevention practice level (KAP) on zoonosis disease, little has been done on livestock producers, farmers, and residents in different areas of the country, both in urban and rural settings. In our study area, the last few years have seen an increase in rural–urban migration, and the majority of migrants have started their businesses in livestock production and the expansion of unplanned areas (slums). In addition, slaughtering animals in unofficial places and selling the product to small butcher shops is common. Buying cattle, slaughtering in unofficial places, and finally shearing by 2–10 people (*kercha* in Amharic) is common. There are inadequate veterinary and public health services for the community. The city is one of the biggest cities and tourist destinations in Ethiopia. All these reasons make it interesting to choose this city. Therefore, assessing the knowledge, attitude, and prevention practice level for the prevention of zoonotic disease and associated factors among animals and animal products is crucial to conducting an evidence-based intervention against zoonotic diseases.

## 2. Materials and Methods

### 2.1. Study Area

**Figure 1:**
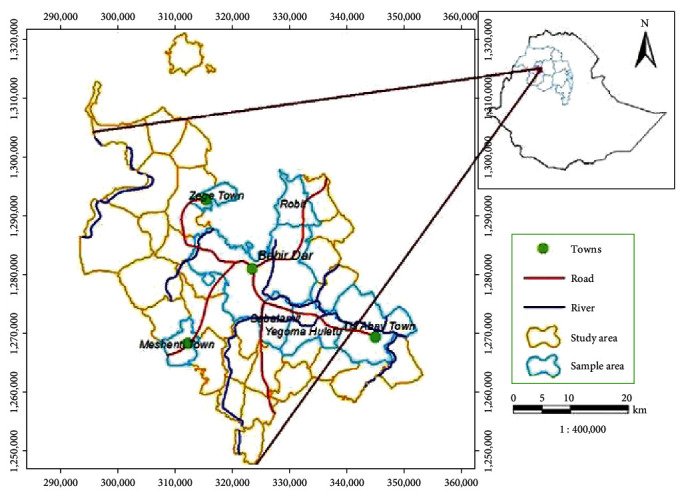
Location map of the study area.

This study was conducted in and around Bahir Dar city. Geographically; it is located where the Blue Nile starts, at 11°29′N latitude and 37°29′E longitude, and situated at 1,799 m above sea level (Figure 1). The region has an average annual rainfall of 850 to 1,250 mm, with daily minimum and maximum temperatures of 10 and 32°C, respectively. The human population of Bahir Dar city is about 455,901 including 222,474 males and 233,428 females [15]. Moreover, 72,833 cattle, 22,149 shoats, and 46,505 poultry were farmed in the study area during the study period [16, 17].

### 2.2. Study Population

The study participants were Bahir Dar city populations comprised of different randomly selected communities, including the different educational levels of farmers, butcher men (an employe or owner actively works as butcher men) and farm attendants such as dairy, beef, and poultry farms in and around Bahir Dar city. The study includes farm owner and farm attendants those actively engaged in farming practices in the city and its surroundings.

Additionally for strengthening and triangulating the findings we include responses from government institutions public health experts (Bahir Dar city administration health office), veterinarians (Bahir Dar city agriculture and livestock office) about Zoonotic Diseases Prevention and Control activities.

### 2.3. Study Design

A questionnaire-based cross-sectional study was conducted from December 2021 to August 2022 to assess the knowledge, attitude, and prevention practice of zoonotic diseases among farm owner and farm attendants those actively engaged in farming practices and their products in Bahir Dar city and its surroundings.

### 2.4. Sampling Method and Sample Size Determination

Before the commencement of the study, preliminary data on livestock farms was sourced from the Bahir Dar city administration's agricultural office. After taking lists of subcities and *kebeles*, livestock producers (dairy, beef, and poultry), and butcher houses were classified based on their farm specialization. Then, from each *kebele*, households having a dairy farm, poultry farm, beef farm, or butcher shop were selected using a cluster sampling method. Moreover, eight institutions were purposely selected to assess their views and interventions on zoonotic diseases in and around Bahir Dar city.

### 2.5. Sample Size Determination

The required sample size for this study was estimated using the single population proportion sample size determination formula [18]. The corresponding statistical parameters considered during the sample size calculation were a 95% confidence interval and 5% absolute precision.

(1) $$\begin{array}{c} n=\frac{{\left(z\right)}^{2}\text{x}P_{\text{exp}}\left(1-P_{\text{exp}}\right)}{d^{2}}, \end{array}$$

where *n* = sample size,

*P*_exp_ = expected prevalence (*P* = 50%),

*Z* = 1.96 for the 95% confidence interval, and

*D* = desired absolute precision.

Considering 50% of the population living in and around Bahir Dar has the required knowledge, attitude, and prevention practice on zoonotic diseases. Thus, the required sample size for this study was estimated at 384. However, after considering 5% of nonresponse rate, a total of 404 individuals were considered as the final sample size for this study.

### 2.6. Inclusion and Exclusion Criteria

Inclusion and Exclusion Criteria: A person (>18 years of age) who resided in a selected household or family and worked on livestock farms and butcher houses were included in the study. Whereas a person unable to provide the necessary information at the time of data collection was excluded from the study.

### 2.7. Data Collection

#### 2.7.1. Data Collection Procedure

A semistructured, pretested questionnaire was used for data collection. The questionnaire was developed after reviewing relevant literature and recent publications. The questionnaire was prepared in English and translated into the local language, Amharic. The questionnaire comprised diverse questions including sociodemographic and livestock characteristics, behavioral, personal, environmental, organizational, knowledge, attitude, and practice of zoonotic disease transmission and prevention.

### 2.8. Variables of the Study

#### 2.8.1. Dependent Variables

For the purpose of this study, knowledge, attitude, and prevention practice were considered dependent on response variables. These variables were classified into two categories (binary scale and good or poor level) to fit the logistic regression analysis.

*(1) Knowledge about Zoonotic Diseases*. The study participant is said to have good knowledge if they can correctly respond with a score greater than or equal to the mean score from those 18 knowledge questions provided. Those respondents who scored below the mean score of 18 knowledge questions were considered poor level of knowledge of zoonotic diseases (mean score from those 18 knowledge questions (50%)) [19, 20].

*(2) Preventive Practice for Zoonotic Diseases*. The study participants were said to have good practice if they could correctly respond greater or equal to the mean score from the 13 practice questions provided [19, 20]. Those respondents who scored below the mean score of 13 practice questions were considered to have a poor level of prevention practices against zoonotic diseases.

*(3) Attitude toward Zoonotic Disease*. Similarly, the study participant was said to have a good attitude if they could correctly respond with a score greater than or equal to the mean score from those nine attitude questions provided.

#### 2.8.2. Independent Variables

Sociodemographic, behavioral, environmental, and personal variables respondents' age, sex, residence location, religion, marital status, educational status, and type of employment (occupation) were defined as sociodemographic variables. Variables including closeness to the media, getting professional training, food consumption practices (raw milk and meat), and caring for the health of animals were considered behavioral variables. The respondent's personal and environmental hygiene and housing system (ventilation and sharing a house with their animals) were considered environmental and personal attributes.

### 2.9. Data Quality Control

Before going to assure data quality, emphasis was given to the design and translation of the questionnaires. The questionnaire was pretested on 20 individuals to ensure its validity and standardize the questionnaire. Frequent checks were made on the data collection process to ensure the completeness and consistency of the gathered information, and errors found during the process were corrected soon.

### 2.10. Data Management and Statistical Analysis

The data obtained from the questionnaire were recorded and coded using a Microsoft Excel spreadsheet, and the data coded in Excel was exported to STATA version 14 for statistical analysis. The results of KAP on zoonotic disease participants' responses were presented using descriptive statistics such as frequency, percentages, and/or mean. A bivariate and multivariable logistic regression analysis was performed to quantify the crude and adjusted effects of the factors on knowledge and practice of zoonosis diseases. Variables with a *p* value of <0.25 in the bivariate analysis were entered into the multivariable analysis. Finally, a *p* value of <0.05 in the multivariable logistic regression analysis was used to identify variables significantly associated with knowledge and practice of zoonotic disease. Both the crude odds ratio (COR) and the adjusted odds ratio (AOR), with 95% confidence intervals, were estimated to show the strength of associations. The Hosmer and Lemeshow test was used to evaluate the goodness of fit for the final multivariable model.

## 3. Results

### 3.1. Socio Demographic Characteristics of Respondents

A total of 404 key respondents (138 dairy, 96 poultry, 96 beef, 38 mixed farms, and 36 butcher shops) were interviewed. The age of participants ranges from 19 to 75, with a mean age of 37.52 years. Of all, 283 (70.05%) of them were male, while 121 (29.95%) of the respondents were female. Regarding their occupational status, 305 (75.5%) of respondents were livestock farm workers on managed dairy, poultry, and beef farms (Table 1).

### 3.2. Attitude Level of Respondents

Of all respondents, about 273 (68.6%) had a good level of attitude. Of all total respondents, about 322 (79.7%) believed that zoonotic disease can be transmitted from animal to human. Most respondents believe that, consumption of raw milk (72.77%) and meat (76.73%) can transmit zoonotic diseases (Table 2).

### 3.3. Knowledge Level of Respondents about Zoonotic Diseases

Of the total 404 study participants, about 365 (90.34%) of the respondents heard about zoonotic diseases, while 9.66% of respondents did not hear about those diseases. The overall respondent's level of knowledge about zoonotic diseases was 52.5% (212/404). Among those who heard about zoonotic diseases, 89% of respondents knew that zoonotic diseases can be transmitted from animal to human, and 27.23% of the respondents knew that zoonotic diseases can be transmitted from human to animal. The majority of respondents believed that dogs (78%), cattle (27%), cats (13.86%), poultry (7.67%), and sheep and goats (6.4%) are important animals that can serve as a source of major zoonotic diseases.

Of the total respondents interviewed, 90.34%, 49.26%, 43.07%, 40.84%, 11.13%, and 3.75% of them were familiar with rabies, bovine tuberculosis, taeniasis, anthrax, brucellosis, and hydatidosis, respectively, and they believed that these diseases are transmitted from animals to humans. Among the total respondents, only 45 (11.14%) of them have received formal training about zoonotic diseases. According to respondent's view, animal bites (79.5%), contact with animals (61.8%), consumption of raw milk and meat (44.6%), and inhalation (37.13%) are important modes of disease transmission (Table 3). Almost half (54.2%) of respondents had heard about zoonotic diseases from their families, and the other 35.9%, 21%, and 11.4% were from school, media, and formal training, respectively.

### 3.4. Prevention Practices of Respondents about Zoonotic Diseases

Among surveyed respondents, only 159 (39.4%) had a good level of prevention practices against zoonotic diseases. Among respondents who owned cattle, about 27.7% of them keep their animals along with their main house hold houses. About 21% of the respondents use hand-protective materials or gloves while performing their routine farm tasks. Nearly two-thirds (59.9%) of respondents bring their animals to veterinary clinics when their animals are sick. About 40.1%, 38.4%, 31.4%, and 29.7% of respondents believed that limiting contact with animals, maintaining hygiene, avoiding consumption of raw food of animal origin, and vaccination were major prevention methods against zoonotic diseases. However, 59.9% and 69.3% of respondents consumed raw milk and meat, respectively (Table 4).

### 3.5. Factors Associated with Knowledge of Respondents for Zoonotic Diseases

Major determinant factors affecting people's knowledge about zoonotic diseases are shown in Table 5. Education status, age, and training were found to be statistically significant with knowledge level in the multivariable logistic regression model. Those respondents who completed their college and university (AOR = 3.71; 95% CI: 1.63, 8.44), secondary (AOR = 3.31; 95% CI: 1.51, 7.28), and primary (AOR = 2.14; 95% CI: 1.14, 4.02) education had a good level of knowledge when compared to those who did not receive a formal education. Likewise, those respondents aged above 50 years (AOR = 3.16; 95% CI: 1.69–4.99) and between 31 and 50 (AOR = 3.09; 95% CI: 1.72–5.53) had a good level of knowledge of zoonotic diseases when compared to younger respondents (Table 5).

### 3.6. Factors Affecting Prevention Practice of People for Zoonotic Diseases

Similar to knowledge responses, respondents' education status, age, and training status were found to be significant factors associated with a good level of preventive practice against zoonotic diseases. Those respondents who took training on zoonotic diseases had a good level of prevention practices (AOR = 4.17, 95% CI: 1.81–12.25) when compared to those who did not take the training. Likewise, those respondents who had completed their primary (AOR = 2.4; 95% CI: 1.15–4.91), secondary (AOR = 3.8; 95% CI: 1.62–8.89), and college and university (AOR = 3.2; 95% CI: 1.35–7.57) education had a good level of prevention practices against zoonotic diseases when compared to those illiterate respondents (Table 6). Likewise, those respondents aged above 50 years (AOR = 3.68; 95% CI: 1.92−7.06) and aged between 31 and 50 (AOR = 2.44; 95% CI: 1.3−4.6) had a good level of prevention practices against zoonotic diseases when compared to younger respondents aged between 19 and 30 years (Table 6).

### 3.7. Responses from Government Institutions about Zoonotic Diseases Prevention and Control

A total of 12 professionals from eight government organizations was included in this study. Of all, 10 (83%) of them were male, and two (17%) of the respondents were female. All respondents believed that the risk of acquiring zoonotic diseases among occupational communities living in and around Bahir Dar city is higher because of the expansion of different intensive livestock farms in and around Bahir Dar city and low level of awareness among livestock producers, animal source food consumers. Among interviewed experts, three (12%) of them had given training about the zoonotic disease to livestock producers in Bahir Dar city.

Most (85%) respondents believed that institutional collaborations were crucial to effectively control and prevent zoonotic diseases, of which 66.7% of them had heard about the role of one health approach in controlling and preventing zoonotic diseases. About 88% of respondents believed that the government had given inadequate attention about zoonotic diseases.

## 4. Discussion

The problem of zoonoses diseases is a global public and veterinary health concern [21]. Knowledge, attitude, and prevention practices and the factors affecting people handling farm animals and their products toward common zoonotic diseases intervention is of interest to numerous research groups recently because of emerging and reemerging of those infections [14, 22–24]. Overall zoonotic pathogens are twice more likely to be associated with emerging disease than nonzoonotic pathogens [25]. Ethiopia is disproportionately at high risks of zoonotic diseases; there are areas characterized by interplay of intense livestock animals and wildlife, coinfection with other diseases, agricultural activities, poor hygiene, unhygienic and insecure food supply, and poor health services [26, 27]. The fact that human health and animal health are indistinguishably linked, global coordinated and well-established interdisciplinary research efforts are essential to successfully fight and reduce the health burden due to zoonoses. This critically requires integrated data from both humans and animals on zoonotic diseases [21, 28].

The present study revealed that 52.5% and 39.4% of respondents had a good level of knowledge and preventive practices about zoonotic diseases, respectively. A relatively similar finding was reported by Hailu et al. [20], who reported that 46.2% and 41.4% of HIV-positive people had a good level of knowledge and practice for bovine tuberculosis in Bahir Dar city, respectively. The slight differences might be attributed to variations in respondents' types and the types of zoonotic diseases studied. For instance, this study covered different respondents drawn from different livestock farms and butcher shops, where major zoonotic diseases other than bovine tuberculosis were addressed.

The current study indicated that 90.34% of the respondents had heard about zoonotic diseases. This finding is supported by the reports of Girma et al. [29] who reported that all respondents found in Addis Ababa had heard about zoonotic diseases. This study recorded that school, family/community, training, and government mainstreaming medias were the major sources of information about zoonotic diseases. A similar finding was reported by Gizachew et al. [30], who reported that people use school, family, and friends as major sources of information about zoonotic diseases in and around the Asella Eastern Arsi Zone, Ethiopia.

It is difficult to establish a universally accepted priority list for zoonotic diseases due to their difference in different areas. Zoonotic diseases vary greatly in their occurrence and impact on human and animal hosts because of variability with the different host, pathogen, and environment related factors [8, 31]. This study revealed that about 90.34%, 40.84%, 43.07%, 40.84%, 11.13%, and 3.71% of respondents believed that rabies, tuberculosis, taeniasis, anthrax, brucellosis, and echinococcosis, can be transmitted from animals to humans, respectively. This study indicated a relatively lower level of awareness among the respondents was recorded in the study area as compared to the report of Tesfaye et al. [6] for rabies (97.1%), followed by taeniasis (83.4%), anthrax (55.4%), bovine tuberculosis (29.1%), and hydatidosis (4%) in Jimma, southwestern Ethiopia. A higher level of awareness among the respondents was also reported by Girma et al. [29], as all respondents (100%) in Addis Ababa mentioned rabies as a zoonotic disease, followed by anthrax (94.27%), taeniasis (89.06%), bovine tuberculosis (88.54%), and brucellosis (49.48%).

In the present study, the majority (89%) of respondents explained that disease can be transmitted from animal to human, while other respondents (27.23%) believed that zoonotic disease can be transmitted from human to animal. This figure was found to be higher when compared to Tesfaye et al. [6], who reported that 15.6% of respondents from the Mana and Limmukosa districts of Jimma zone believed that zoonotic diseases can be transmitted from humans to animals. It was also found higher when compared to Barnes et al. [32] among Mongolian herding households, who reported that most households had knowledge about zoonotic disease transmission (74%), but far less recognized the risk of zooanthroponosis or human-to-animal disease transmission (53.3%). The difference in overall awareness among different studies for the common zoonotic diseases could be due to variations in educational status, exposure to information sources, and living styles of people between study areas.

The majority of respondents believed that dogs (78%), cattle (27%), poultry (7.67%), sheep and goats (6.4%), and cats (13.86%) are important animals that can serve as a source of major zoonotic diseases. A relatively similar finding was reported by Syidul and Ahmed [33] in Barguna district, Bangladesh, reported the highest percentage of respondents in the reported study mentioned that dog (52.99%) is the most important source of the transmission of zoonotic disease followed by cats (28.87%), poultry (28.25%), cattle/buffalo (16.085%), and sheep/goat (9.90%).

Regarding the food consumption habits of respondents, 59.9% and 69.31% of respondents consume raw milk and meat, respectively. This finding was lower than the reports of Hailu et al. [20], who reported that 36.8% and 9.2% of the respondents consume raw milk and meat, respectively, in Bahir Dar city. However, a similar finding was reported by Tesfaye et al. [6], who reported that about 58.2% and 57.1% of respondents in Mana and Limmukosa districts, Ethiopia, consumed raw meat and milk, respectively. Raw meat consumption was also reported by a large proportion of respondents (69.1%) in Jimma, southwestern Ethiopia, Tesfaye et al. [6]. This revealed that there was a deep-rooted culture of raw meat consumption in Ethiopia in general and in the present study area in particular. The slight discrepancy between the present and previous studies might be associated with variations in food consumption behavior among surveyed respondents.

The practice of consuming raw meat, raw milk, and its products is attributed to a lack of awareness about zoonoses that bring behavioral change and/or negligence [34, 35]. Moreover, it has been reported that a lack of knowledge of zoonosis combined with food consumption habits and poor animal husbandry is likely to expose respondents to an increased risk of contracting zoonosis. The predictors of knowledge and practice in this study were education, age, training, and type of farm. The present study revealed that those respondents who attended college and above had a good level of knowledge and practice about zoonotic diseases. The possible explanation could be that an educated person would have better access to information and could easily understand the disease. Similar findings were reported by Deneke et al. [35]. in urban and peri-urban dairy farmers in Ethiopia and Ismaila et al. [36] in Nigeria. Providing education plays an important role in adding to knowledge and also in increasing zoonosis disease awareness among the owners of livestock [37].

Those respondents aged above 50 years and between 31 and 50 years had good knowledge and practice levels as compared to younger people. This finding was consistent with other previous reports in Nigeria [36], which reported that respondents in the age group >58 years had high knowledge. However, this finding is different from the previous report in Bahir Dar [20], which reported that respondents in the age group between 46 and 60 years had lower knowledge than those younger. The statistically significant difference *p* < 0.05 among age groups might be due to variations in experiences among age groups, where older people have better experience and awareness about zoonotic diseases.

In the present study, of the total respondents who got information about zoonotic diseases, only 11.4% got training about zoonotic diseases. This supports interviews done by professionals. About 3 (12%) respondents took and gave training about the zoonotic disease. This decreases the number of training professionals and farm owners due to the absence of a policy for zoonotic disease prevention and control and the absence of a budget. Lack of interdisciplinary training for veterinarians and human physicians hinders collaboration in the intervention of zoonotic diseases. Policymakers need to move beyond rhetoric and really focus on health care reform and the implementation of policies that link human, animal, and environmental health. We need interdisciplinary and multidisciplinary training on human, animal, and environmental health, and collaborative research on the prevention of zoonotic diseases [38].

Creating networks and improving communication within and between sectors, locating points of agreement to coordinate around and spotting opportunities to get going, acknowledging that integration can occur at various levels and won't always be appropriate, allocating resources fairly and investing in prevention at the source, leading change while recognizing and encouraging individual contributions in zoonotic disease prevention is paramount important [38, 39].

## 5. Conclusion and Recommendations

The present study revealed that 52.5%, 68.6%, and 39.4% of respondents had a good level of knowledge, attitudes, and preventive practices about zoonotic diseases, respectively. This indicates that the overall level of attitude, knowledge, and practice among the occupational community in and around Bahir Dar city on common zoonosis diseases was found low. A substantial proportion of respondents were consuming raw milk and meat in the study areas, which could play a significant role in the transmission of zoonotic diseases. Survey findings revealed that rabies, tuberculosis, taeniasis, anthrax, and brucellosis were the major zoonotic diseases identified by respondents. Limiting contact with animals, maintaining good personal and environmental hygiene, and avoiding consumption of raw food of animal origin were the major prevention methods against zoonotic diseases. Dogs, cattle, poultry, sheep, goats, and cats are the major livestock types that can serve as sources of major zoonotic diseases. The respondent's education status, age, farm location, and training status were found to be significant risk factors associated with a good level of knowledge and prevention practices against zoonotic diseases. Therefore, developing and applying interventions against the identified risk factors is highly required to minimize the effect of zoonotic diseases in the study areas. Besides, awareness-raising and raising livestock producers' awareness about the transmission methods and impacts of the zoonotic disease are suggested. Consumption of raw milk and meat should be discouraged. More importantly, the application of one health approach is crucially important to control zoonotic diseases, and a concerted effort among all relevant stakeholders, especially among human and animal health professionals is highly needed to effectively control and prevent zoonotic diseases. Further research on the economic and public health impacts of zoonotic disease is suggested in the study area.

## Figures and Tables

**Table 1:** Sociodemographic characteristics of the respondents in and around Bahir Dar city.

Characteristics	Frequency	%
Sex		
Male	283	70.05
Female	121	29.95
Age (in years)		
19–30	122	30.2
31–50	159	39.36
>50	123	30.45
Religion		
Orthodox	369	91.34
Muslim	35	8.66
Marital status		
Single	99	24.50
Married	305	75.50
Educational level		
Illiterate	96	23.76
Primary (1–8)	169	41.83
Secondary (9–12)	66	16.34
College/university	73	18.07
Occupation status		
Mixed farming	38	9.41
Livestock farm worker	330	81.68
Butcher	36	8.91
Residence		
Urban	286	70.79
Peri-urban	118	29.21

**Table 2:** Participant's response to attitude questions among people handling farm animals and their products.

Attitude variables	Category	Number of respondents	Percentage (%)
Man contact with animal infected by zoonotic diseases	Agree	327	81.25
	Disagree	77	18.75

Raw milk consumption can be a source of infection for zoonotic diseases	Agree	294	72.77
	Disagree	110	27.23

Zoonotic diseases restrict international trade	Agree	327	81.25
	Disagree	77	18.75

Raw meat consumption can be a source of infection for zoonotic diseases	Agree	310	76.73
	Disagree	94	23.27

Access to media can be helpful to create or raise awareness about zoonotic diseases	Agree	325	80.44
	Disagree	79	19.56

Working with others sectors can helpful to prevent zoonotic diseases	Agree	322	79.7
	Disagree	82	20.3

**Table 3:** Knowledge related responses among people handling farm animals and their products.

Knowledge questions	Response	Frequency	Percentage (%)
Hear about zoonotic diseases	Yes	365	90.34
	No	39	9.6

Type zoonotic diseases they heard/knew	Rabies	365	90.34
	Tuberculosis	154	49.26
	Anthrax	165	40.84
	Taeniasis	174	43.07
	Brucellosis	45	11.13

Zoonotic diseases are transmitted	Hydatidosis	15	3.71
	Animal bite	321	79.45
	Contact	250	61.8
	Consumption of animal origin food	180	44.5
	Inhalation	150	37.13
	Others	35	8.6
	I do not know	25	6.1

**Table 4:** Participant's response to practice questions people handling farm animals and their products in and around Bahir City.

Questions for prevention practices	Category	Number of respondents	Percentage (%)
Drink raw milk	Yes	242	59.90
	No	162	40.1

Eat raw meat	Yes	280	69.31
	No	124	30.69

Live together with animals	Yes	120	29.7
	No	284	70.3

Use personal protective equipment	Yes	85	21
	No	319	79

Prevent zoonotic disease	Vaccination	120	29.7
	Limit contact	158	40.1
	Keeping hygiene	155	38.4
	Avoid consumption of raw food of animal origin	127	31.4
	No	80	19.8

Cattle infected with zoonotic diseases	Veterinary clinic	242	59.9
	Isolation	120	29.7
	Traditional healer	83	20.54

Person infected with zoonotic diseases	Medical healer	234	84.16
	Traditional healer	170	42.07
	Others	85	21.03

**Table 5:** Multivariable logistic regression of factors affecting the knowledge of people in and around Bahir city.

Variables	Knowledge level		COR (95% CI)	AOR (95% CI)
	Good	Poor		
Education				
Illiterate	26	70	−Ref	−Ref
Primary (1–8)	89	80	2.99 (1.74–5.15)	2.14 (1.14–4.02)
Secondary (9–12)	42	24	4.71 (2.40–9.24)^a^	3.31 (1.51–7.28) ^*∗*^
College and university	55	18	8.23 (4.1–16.52)^a^	3.71 (1.63–8.44) ^*∗*^
Age				
19–30	40	82	Ref	Ref
31–50	88	71	2.54 (1.56–4.15)^a^	3.09 (1.72–5.53)
>50	44	39	4.42 (2.58–7.55)^a^	3.16 (1.69–5.90) ^*∗*^
Residence				
Urban	176	110	3.64 (2.3–5.76)^a^	2.34 (1.32–4.14) ^*∗*^
Peri-urban	36	82	Ref	Ref
Training				
Yes	41	4	11.27 (3.95–32.11)^a^	3.79 (1.23–11.69) ^*∗*^
No	171	188	Ref	Ref
Type of farm				
Beef	24	72	Ref	Ref
Dairy	76	62	3.68 (2.08–6.50)^a^	4.14 (2.18–8.89)
Mixed	17	21	2.43 (1.1–5.35)^a^	4.37 (1.75–10.89)
Poultry	78	18	12.99 (6.525.91)^a^	7.1 (3.4–15.21)

CI = confidence Interval, COR = crude odds ratio, and AOR = adjusted odds ratio.  ^*∗*^ = significant at 5% (*p*  < 0.05), ^*a*^ = significant at 25% (*p* < 0.25); and Ref = reference.

**Table 6:** Multivariable logistic regression of factors affecting the practice of people.

Variables	Level of prevention practices		COR (95% CI)	AOR (95% CI)
	Good	Poor		
Education				
Illiterate	14	82	Ref.	Ref
Primary (1–8)	67	102	3.85 (2.02–7.33)^a^	2.37 (1.15–4.91)
Secondary (9–12)	34	32	6.22 (2.96–13.1)^a^	3.8 (1.62–8.89) ^*∗*^
College/university	44	29	8.89 (4.26–18.54)^a^	3.2 (1.35 −7.57) ^*∗*^
Age				
19–30	27	95	Ref	Ref
31–50	60	99	2.13 (1.25–3.64)^a^	2.44 (1.3–4.6)
>50	72	51	4.97 (4.25–18.5)^a^	3.68 (1.92–7.06) ^*∗*^
Residence				
Urban	137	149	4.01(2.39–6.74)^a^	2.33 (1.32–4.14) ^*∗*^
Peri-urban	22	96	Ref	Ref
Training				
Yes	39	6	12.94 (5.33–31.43)^a^	4.17 (1.81–12.25) ^*∗*^
No	239	120	Ref	Ref
Farm type				
Poultry	68	28	6.5 (3.01–14.04)^a^	5.88 (2.78–12.46)
Dairy	50	88	2.71 (1.33 5.52)^a^	2.53 (1.25–5.09)
Mixed	14	24	4.96 (.814 13.60)^a^	5.37 (1.99–14.47)
Beef	17	79	Ref	Ref

CI = confidence interval, COR = crude odds ratio, AOR = adjusted odds ratio, ^*a*^ = significant at 25% (*p* < 0.25),  ^*∗*^ = significant at 5% (*p* < 0.05), and ref = reference.

## Data Availability

All information is available upon reasonable request from the corresponding author.
